# Automated hearing loss type classification based on pure tone audiometry data

**DOI:** 10.1038/s41598-024-64310-2

**Published:** 2024-06-20

**Authors:** Michał Kassjański, Marcin Kulawiak, Tomasz Przewoźny, Dmitry Tretiakow, Jagoda Kuryłowicz, Andrzej Molisz, Krzysztof Koźmiński, Aleksandra Kwaśniewska, Paulina Mierzwińska-Dolny, Miłosz Grono

**Affiliations:** 1 Department of Geoinformatics, Faculty of Electronics, Telecommunications and Informatics, Gdańsk University of Technology, G. Narutowicza 11/12, 80-233 Gdańsk, Poland; 2 Department of Otolaryngology, Medical University of Gdańsk, Gdańsk, Poland; 3 Department of Otolaryngology, The Nicolaus Copernicus Hospital in Gdańsk, Copernicus Healthcare Entity, Gdańsk, Poland; 4 Department of Otolaryngology, Laryngological Oncology and Maxillofacial Surgery, University Hospital No. 2, Bydgoszcz, Poland

**Keywords:** Classification, Bi-LSTM, Hearing loss, Tonal audiometry, Audiogram, AI decision support system, Health care, Medical research, Engineering

## Abstract

Hearing problems are commonly diagnosed with the use of tonal audiometry, which measures a patient’s hearing threshold in both air and bone conduction at various frequencies. Results of audiometry tests, usually represented graphically in the form of an audiogram, need to be interpreted by a professional audiologist in order to determine the exact type of hearing loss and administer proper treatment. However, the small number of professionals in the field can severely delay proper diagnosis. The presented work proposes a neural network solution for classification of tonal audiometry data. The solution, based on the Bidirectional Long Short-Term Memory architecture, has been devised and evaluated for classifying audiometry results into four classes, representing normal hearing, conductive hearing loss, mixed hearing loss, and sensorineural hearing loss. The network was trained using 15,046 test results analysed and categorised by professional audiologists. The proposed model achieves 99.33% classification accuracy on datasets outside of training. In clinical application, the model allows general practitioners to independently classify tonal audiometry results for patient referral. In addition, the proposed solution provides audiologists and otolaryngologists with access to an AI decision support system that has the potential to reduce their burden, improve diagnostic accuracy, and minimise human error.

## Introduction

Hearing is a key sense in human daily existence, allowing for connectivity with the outside world in a manner that none of our other senses can accomplish. Aside from enabling efficient communication with others, good hearing is crucial for personal safety, e.g. when crossing the street on foot, operating a vehicle, or responding to a fire alarm, frequently enabling detection of a potential threat before it becomes visible. Other benefits that good hearing may bring to quality of life, such as listening to music, television and radio, also should not be overlooked. Extreme cases of communication difficulties, resulting in a decline in quality of life, may lead to psychiatric disorders such as depression^1^.

According to the World Health Organization (WHO), hearing loss currently affects more than 1.5 billion people worldwide, of whom 430 million have moderate or higher levels of hearing loss in the better hearing ear. WHO predicts that by 2050, nearly 2.5 billion people will have some degree of hearing loss, with at least 700 million requiring rehabilitation services. Fortunately, early detection and efficient management can significantly mitigate numerous instances of hearing impairment, particularly those associated with childhood hearing loss. Medical and surgical methods can be effective in the treatment of ear diseases, in many cases leading to restoration of original hearing quality^2^.

Hearing loss is predominantly determined with the use of pure-tone audiometry, typically performed while seated in a sound-proof chamber. It involves delivering a series of increasingly-intense pure tones at predetermined threshold levels, typically via headphones, and determining the auditory threshold for air and bone conduction. Air conduction determines the function of the complete auditory organ, from the auricle to the temporal lobe hearing centres. Any level of damage to this system decreases the air conduction curve. Bone conduction examines the organ of hearing from the level of the bony capsule of the cochlea, bypassing the conduction of sound through the outer and middle ear. It serves as an alternative pathway for sound conduction, although it is not as significant as air conduction. Using pure-tone audiometry, which assesses both air and bone conduction, it is possible to identify the type of hearing impairment. Conductive hearing loss is usually caused by diseases of the external auditory canal and/or middle ear. Sensorineural hearing loss occurs due to damage to the sensory cells and/or nerve fibers of the inner ear^3^. Mixed hearing loss is the result of both sensorineural and conductive hearing loss^4^. Hearing loss can be unilateral or bilateral, sudden or chronic, and can range in severity from mild to profound. Hearing impairment is common, particularly among patients with aural disease and the elderly.

The majority of hearing losses in clinical populations are sensorineural and mixed^5^. While the sensorineural components are rarely curable, accurate diagnosis is a major impediment in successful treatment. Audiologists, who are necessary for proper execution and interpretation of tests, are in short supply globally. Among low-income countries, in particular, approximately 93% have fewer than one audiologist per million. Even in nations with relatively high numbers of practitioners in the field of ear and hearing care, inequitable distribution and other factors can limit access to these specialists^2^. Artificial intelligence (AI) has the potential to address this issue, given the disparity between the supply and demand for hearing specialists. AI implements algorithms that allow computers to recognize specific data analysis patterns and draw conclusions. In the healthcare industry, this software analyses human cognition to establish links between various types of treatments and the subsequent medical outcomes. One of the most common uses of machine learning in medicine is the analysis of images such as computed tomography (CT) and magnetic resonance imaging (MRI) to detect various types of irregularities, including tumours, ulcers, fractures, internal bleeding etc., in order to provide crucial data for health care specialists and their patients. As a result, AI assists radiologists in automating daily administrative duties, improves diagnostic accuracy, eliminates human error risks, and allows researchers to concentrate on complex cases^6,7^. This is also true in tonal audiometry, where AI has been applied to the determination of edge frequency of a high-frequency dead zone in the cochlea as well as to assistance in fine-tuning hearing aids to the client’s preferences more precisely and efficiently^8^.

In the above context, this paper proposes a neural network model for classification of hearing loss types for discrete tonal audiometry data series. The primary objective was to obtain classification accuracy sufficient for clinical application of the developed network, allowing general practitioners to classify tonal audiometry results autonomously for further patient referral. This could result in lessening the burden on audiology specialists while still ensuring that the final decision on diagnosis is made by a physician. For audiologists, the system might eliminate simple cases, allowing them to concentrate on the more complex ones, as well as enhance diagnostic precision and prevent human error in daily practice. Furthermore, the aim is to exceed the current state-of-the-art in classification of raw audiometry data, which currently achieves an accuracy rate of 95.5% through the application of Decision Trees^9^.

The paper is structured as follows: the second section describes the materials and techniques used to train and evaluate the classification model. Specifically, types of hearing loss are described in section “Hearing loss types”, a literature review can be found in section “Automatic classification of audiometry data”, the dataset is described in section “The tonal audiometry dataset”, ethics declarations are included in section “Ethics declarations” and the study methodology is explained in section “Methodology”. The third section provides detailed results. The fourth section discusses the obtained results and their comparison to the current state-of-the-art. Finally, the fifth section presents the conclusions.

## Materials and methods

### Hearing loss types

According to the WHO, hearing loss may be classified as conductive, sensorineural or mixed^2^. In conductive hearing loss, lesions develop in the conductive component (outer and middle ear). This type of hearing loss is characterised by good telephone speech comprehension, better hearing and speech discrimination in noise than in silence, improved speech comprehension after amplification, and preserved voice control. Conductive hearing loss may be diagnosed on the basis of audiometric tests and otoscopy. In tonal audiometry, it is characterised by a normal bone conduction, a lowering in the air conduction curve, and the presence of an air–bone gap, i.e. a 15–40 dB difference between the bone and air curves. Congenital deformities of the external and middle ear, otitis externa and otitis media, otosclerosis, injury of the external auditory canal, wax plug, obstruction of the auditory tube, and tumours of the temporal bone and nasopharynx are the most common causes of conductive hearing loss^10^.

Sensorineural hearing loss is a hearing impairment induced by inner ear and auditory nerve disorders. It can be cochlear—caused by damage to the organ of Corti—or extracochlear—affected by damage to the auditory nerve up to the cochlear nuclei. The characteristics of sensorineural hearing loss are: better hearing through the air, impaired understanding of speech in noise, better hearing of low sounds, unpleasant perception of high sounds, different perception of sounds in both ears. On audiometric examination, bone and air conduction curves are at the same level, and there is no air–bone gap. The most common causes of sensorineural hearing loss with cochlear localization are: hearing loss caused by ageing, acute and chronic acoustic trauma, congenital defects, skull base fractures, pressure barotrauma, ototoxic drugs, chemotherapy, labyrinthitis, vascular disorders of the inner ear, Ménière's disease, cochlear otosclerosis, radiotherapy and metabolic disorders. Causes of sensorineural hearing loss with extracochlear and central location include presbycusis, multiple sclerosis, cranial trauma and fractures, meningitis, cerebello-pontine angle tumours, brain tumours and cerebrovascular diseases^11^.

Mixed hearing loss is a combination of sensorineural and conductive hearing loss in a single ear. It may be the result of a single disease, such as otosclerosis or suppurative otitis media, or of the superimposition in one ear of two or more of the diseases listed above. It is characterised by a decreasing of the auditory threshold for bone conduction and air conduction with the existence of air–bone gap, impaired speech comprehension dependent on the sensory-nervous component, and an audiometric curves demonstrating less decreasing in the low tones and more greater decreasing in the high tones.

Hearing impairment, particularly sensorineural hearing loss, is prevalent among the elderly and tends to aggravate with age. Conductive hearing loss is typical of adolescents and adults of working age; if it worsens, it does so very gradually, as in otosclerosis.

The described forms of hearing loss are treated differently. In the conductive type, surgical treatment predominates: paracentesis, ventilation tube placement, myringoplasty, and tympanoplasty. The majority of cases of sensorineural hearing loss are treated conservatively, as they result from sudden deafness, acute acoustic trauma and multiple sclerosis. In those instances, rehabilitation with the use of a hearing aid often proves effective. In specific cases, hearing rehabilitation must be combined with surgical treatment, as with cochlear implants. Mixed hearing loss is treated based on its aetiology. Stapedotomy is the surgical treatment for otosclerosis, whereas in non-surgical cases hearing devices are fitted^4,12^.

#### Data selection criteria

The inclusion and exclusion criteria for tonal audiometry data in the dataset were determined according to rules given by Margolis and Saly^13^. During the initial stage of the classification process, every audiometry test result was evaluated to determine if it met the minimum standards for inclusion in this study. Initially, a thorough examination was performed to verify the existence of six octave frequencies, namely 250 Hz, 500 Hz, 1000 Hz, 2000 Hz, 4000 Hz and 8000 Hz in air conduction. In the context of bone condition, the auditory thresholds for four specific octave frequencies (500 Hz, 1000 Hz, 2000 Hz, 4000 Hz) were examined to determine their presence. If any of these values were absent, the data was rejected. Furthermore, any audiometry test result that satisfied any of the following criteria was also eliminated:

- Air conduction threshold is outside the range of − 10 to 110 dB HL (exceeding 250 Hz, when the limit is 90 dB HL);
- Air conduction threshold is beyond the 0–30 dB range of the next lower frequency.
- Bone conduction threshold is beyond the range of − 10 to 60 dB HL (exceeding 250 Hz, when the limit is 40 dB HL);
- The bone-conduction threshold should fall within the range of 50 to 10 dB relative to the air-conduction threshold at that frequency.

#### Classification of hearing loss types

Based on the latest (2021) WHO standards^2^, normal hearing is defined as an average value for the air and bone conduction curve evaluated at 4 octave frequencies (0.5 kHz, 1 kHz, 2 kHz, 4 kHz) that is below 20 dB HL. These guidelines are also in line with the 1996 International Bureau for Audiophonology criteria^14^. The air-bone reserve, sometimes referred to as the air–bone gap, was calculated by subtracting the individual values of frequencies in the air-bone conduction threshold for in 500 Hz, 1000 Hz, 2000 Hz and 4000 Hz. The present air-bone reserve was determined to be 10 dB HL at three or more frequencies within the range of 500–4000 Hz or 15 dB HL for single frequency within this range^13^. The diagnosis of conductive hearing loss was made based on the existence of hearing loss in the air conduction curve, normal values in the bone conduction curve and the presence of air-bone reserve. The identification of sensorineural hearing loss was determined based on the observation of hearing loss in both the air and bone conduction curve as well as the absence of air-bone reserve. The medical diagnosis of mixed hearing loss has been established by noticing hearing loss in both the air and bone conduction curves, in addition to the presence of air-bone reserve. Table 1 provides a comprehensive list of the specific criteria that were utilized to classify the audiograms.

**Table 1:** Classification criteria of hearing loss types.

	Air condition	Bone condition	Air–bone gap
Normal hearing	average value for frequencies of 500–4000 Hz < 20 dB	average value for frequencies of 500–4000 Hz < 20 dB	absence of an air–bone gap
Conductive hearing loss	average value for frequencies of 500–4000 Hz ≥ 20 dB	average value for frequencies of 500–4000 Hz < 20 dB	10 dB across minimum three frequencies (500–4000 Hz) or 15 dB across one frequency (500–4000 Hz)
Sensorineural hearing loss	average value for frequencies of 500–4000 Hz ≥ 20 dB and average value for frequencies of 4000–8000 Hz ≥ 20 dB	average value for frequencies of 500–4000 Hz ≥ 20 dB and average value for frequencies of 4000–6000 Hz ≥ 20 dB	absence of an air–bone gap
Mixed hearing loss	average value for frequencies of 500–4000 Hz ≥ 20 dB	average value for frequencies of 500–4000 Hz ≥ 20 dB	10 dB across minimum three frequencies (500–4000 Hz) or 15 dB across one frequency (500–4000 Hz)

### Automatic classification of audiometry data

In medical practice, the type of hearing impairment is determined from pure-tone audiometry test results according to their configuration, severity, location of lesion (hearing loss type) and symmetry^13,15^. The process is performed by audiology specialists on the graphical representation of an audiometry test result, known as an audiogram. The site of lesion is determined by air and bone conduction thresholds of the audiogram, whereas the configuration is determined by shape. The severity is determined by the degree of hearing loss.

The subject of automatic audiometry data classification has been under investigation for a long time. Over the last decade, there have been a number of attempts at devising an automated method of classification that would be accurate enough to warrant practical application.

The first attempt in this regard was made by Cheng-Yung Lee et al.^16^, who proposed a statistical classification system of audiogram shapes in an effort to enhance and integrate shape recognition across clinical settings. Based on 1633 audiograms, eleven audiometric shapes were classified using K-means cluster analysis. The authors anticipated that, in the future, the classification of audiogram shapes would result in a more efficient infrastructure for diagnosing hearing loss.

Further work may be divided into two thematic groups: classification of audiogram shapes for the purpose of determining the initial configurations of hearing aids^17,18^ and diagnosing the type of hearing loss.

Chelzy Belitz et al.^19^ combined unsupervised and supervised machine learning techniques for mapping audiograms to a limited number of hearing aid configurations. When mapping a single configuration to each audiogram, the best results were achieved with the Multi-layer Perceptron model at 64.19% accuracy. When mapping two configurations to each audiogram, the chance that at least one is correct increased to 92.70%.

Charih et al.^20^ presented their Data-Driven Annotation Engine, a decision tree based audiogram multi-label classifier which considers the configuration, severity and symmetry of participant’s hearing losses and compared it to AMCLASS^13^, which fulfils the same purpose using a set of general rules. Dataset used in this study contained 270 distinct audiograms with seven tested frequencies at 500 Hz, 1,000 Hz, 2,000 Hz, 3,000 Hz, 4,000 Hz, 6,000 Hz and 8,000 Hz. However, bone conduction information is not included in the data set. Three licensed audiologists rated the method's accuracy at approximately 90 percent.

Abeer Elkhouly et al.^21^ proposed a machine learning solution to classify audiograms for the purpose of configuring hearing aids based on their shapes using unsupervised spectral clustering, normalization, and multi-stage feature selection on a dataset of 28 244 audiograms. The authors normalized the data using 20 different normalization methods to increase the training data size in building a credible model, and then selected 10 normalized data sets to train the model. Firstly, the data was divided into 10 clusters, then classified using fine K nearest neighbour classifier with 95.4% accuracy.

In comparison to the subject of automated configuration of hearing aids, the problem of hearing loss type classification has been given considerably less attention.

In this regard, Elbaşı and Obali^9^ presented a comparison of several approaches to hearing loss determination, including Decision Tree C4.5 (DT-J48), Naive Bayes and Neural Network Multilayer Perceptron (NN) model. The study was conducted on a dataset containing 200 samples divided into four categories, including normal hearing, conductive hearing loss, sensorineural hearing loss, and mixed hearing loss. Input data was formatted as a series of numeric values representing Decibels corresponding to constant frequency levels (750 Hz, 1 kHz, 1.5 kHz, 2 kHz, 3 kHz, 4 kHz, 6 kHz, 8 kHz). Classification algorithms have been implemented using Weka software, resulting in 95.5% accuracy in Decision Tree, 86.5% accuracy in Naive Bayes, and 93.5% accuracy in NN.

More recently, Crowson et al.^22^ adopted the ResNet models to classify audiogram images into three types of hearing loss (sensorineural, conductive or mixed) as well as normal hearing using a set of training and testing images consisting of 1007 audiograms. The model was fed by 500 × 500 pixel images of static audiogram plots that had been pre-transformed. Instead of fully training the classifier, the authors applied transfer learning to well-established raster classification models. All tested architectures were based on convolutional neural network (CNN) architectures, but the ResNet-101 model achieved the highest classification accuracy at 97.5%.

In conclusion, the integration of neural networks with enhanced computational capabilities and more extensive training datasets should enable more comprehensive evaluations^23^. Despite this, the classification accuracy of the majority of the currently proposed solutions ranges between 90 and 95%, which, while very high, still leaves substantial room for error. According to clinical standards, the margin of error should be kept under 5%^24^ and ideally should be close to 3%^25^. Only one of the discussed classifiers satisfies these requirements. Crowson et al.^22^ presented the finest audiogram classifier to date, using transfer learning to adapt an established image classifier network to the analysis of audiogram images. Despite producing a classification accuracy of 97%, this method has significant limitations. Due to the fact that it is an image classifier, it cannot be applied to the original data series generated by tonal audiometry. This necessitates converting the data series into audiogram images, which may result in data loss. Moreover, although the structure of audiograms is generally similar, audiograms generated by distinct hardware and software configurations can still differ significantly. In addition to differences in background and line colours, audiograms can also differ in the amount of information conveyed (e.g. they may contain data for a single ear or both). Consequently, a universal classification solution for tonal audiometry results cannot rely on an image classifier^26^.

In addition, each of the cited studies on determining hearing loss type was conducted with a relatively small data set, ranging from 200 test results in Elbaşı and Obali^9^ to 1007 in Crowson et al.^22^, which may have led to an optimistic and unreliable estimation of model performance. Moreover, the limited size of the training dataset poses a challenge in discerning relationship patterns within certain classes, potentially leading to a validation outcome that has bias when applied to the test dataset.

In the above context, a summary of the current state-of-the-art is presented in Table 2.

**Table 2:** Overview of state-of-the-art methods and results.

Paper	Audiogram classification problem	Data size	Data type	Accuracy (%)
Cheng-Yung Lee et al.^16^	Configuration—11 shapes	1633	Raster/raw data	–
Chelzy Belitz et al.^19^	Configuration—4 shapes	90 000	Raster/raw data	64
Charih etc.^20^	Configuration—8 shapes, severity, and symmetry	320	Raster data	90
Abeer Elkhouly et al.^21^	Configuration—10 shapes	28 244	Raster/raw data	95
Ersin Elbaşı and Murat Obali^9^	Hearing loss types: normal, conductive, mixed and sensorineural	200	Raw data	95.5
Crowson etc.^22^		1007	Raster data	97.5

As can be seen in Table 2, thus far the issue of hearing loss type classification has been researched on relatively small data samples with undisclosed class ratios, and the best achieved classification accuracy has been produced for a raster dataset, resulting in limited application. Consequently, the presented work focused on the development of a classifier trained on a considerably larger and more representative data set. Moreover, the developed classifier has been designed for use with raw audiometry data, ensuring greater flexibility of application.

### The tonal audiometry dataset

**Figure 1:**
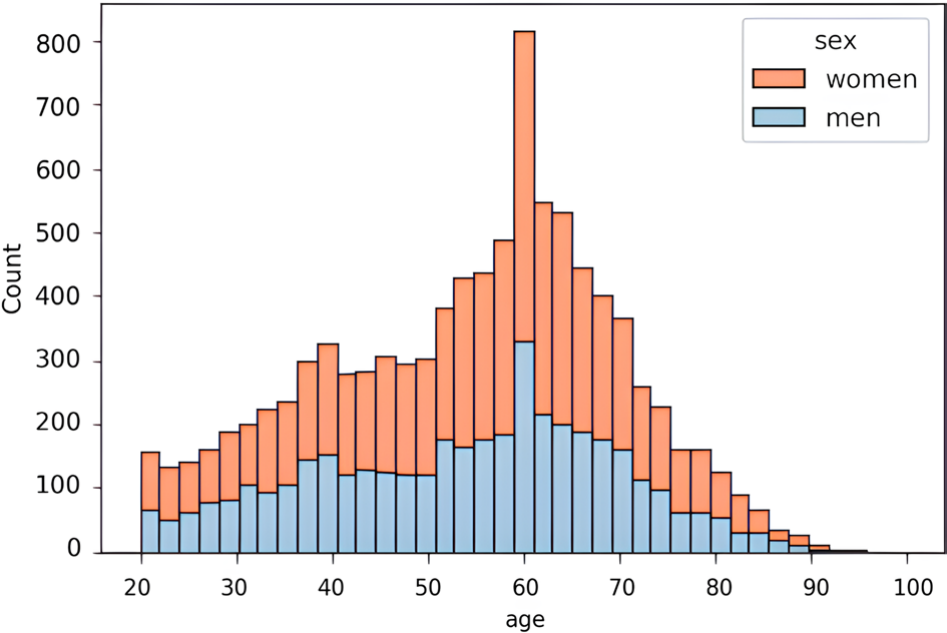
The distribution of patients’ age and sex in the dataset.

The dataset includes 15,046 audiometry test results from 9663 adult patients tested between 2010 and 2022 in the Otolaryngology Clinic of the University Clinical Centre in Gdansk, Poland. Tonal audiometry tests were conducted in soundproof booths (ISO 8253, ISO 8253). Signals were generated by calibrated Itera II and Midimate 622 clinical audiometers, manufactured by Madsen Electronics (Otometrics, Copenhagen, Denmark) (PN-EN 60645-1, ISO 389, ISO 8789, ISO 7566, ISO 8798). The equipment had the ability to correct for ANSI S 3.6-1989 and 2004 standard hearing levels. The American Speech-Language-Hearing Association (ASHA) guidelines were used in the evaluation of participants' hearing by tonal audiometry^27^. Using air conduction tests, the signal generated by the audiometer was connected to TDH-39P headphones. For bone conduction tests, the audiometer was coupled to a B-71 bone vibrator (New Eagle, PA). Of the examined patients, aged between 18 and 98, 5591 were female (57.86%) and 4072 were male (42.14%). The patients’ age distribution by sex has been illustrated in Fig. 1. A maximum of two test results were obtained from each patient, one for the left ear and one for the right, resulting in no replication of data from the same patient and ensuring good data variety.

Three experienced audiologists labelled the morphologies of hearing loss on the audiometry test results, dividing the set into four classes: normal hearing, conductive hearing loss, mixed hearing loss, and sensorineural hearing loss according to methodology presented in Table 1. The evaluation of every test results was conducted by three audiologists. In cases where the classification result was not unanimous, the final decision was made by majority vote in which the highest weight was given to the opinion of the senior audiologist (T.P.). Table 3 shows the quantity of samples for each class in the produced dataset.

**Table 3:** The four hearing types contained in the dataset and the number of samples in each group.

Hearing type	Number of samples (%)
Normal	2584 (17.17%)
Conductive hearing loss	657 (4.37%)
Mixed hearing loss	4028 (26.71%)
Sensorineural hearing loss	7777 (51.69%)

**Figure 2:**
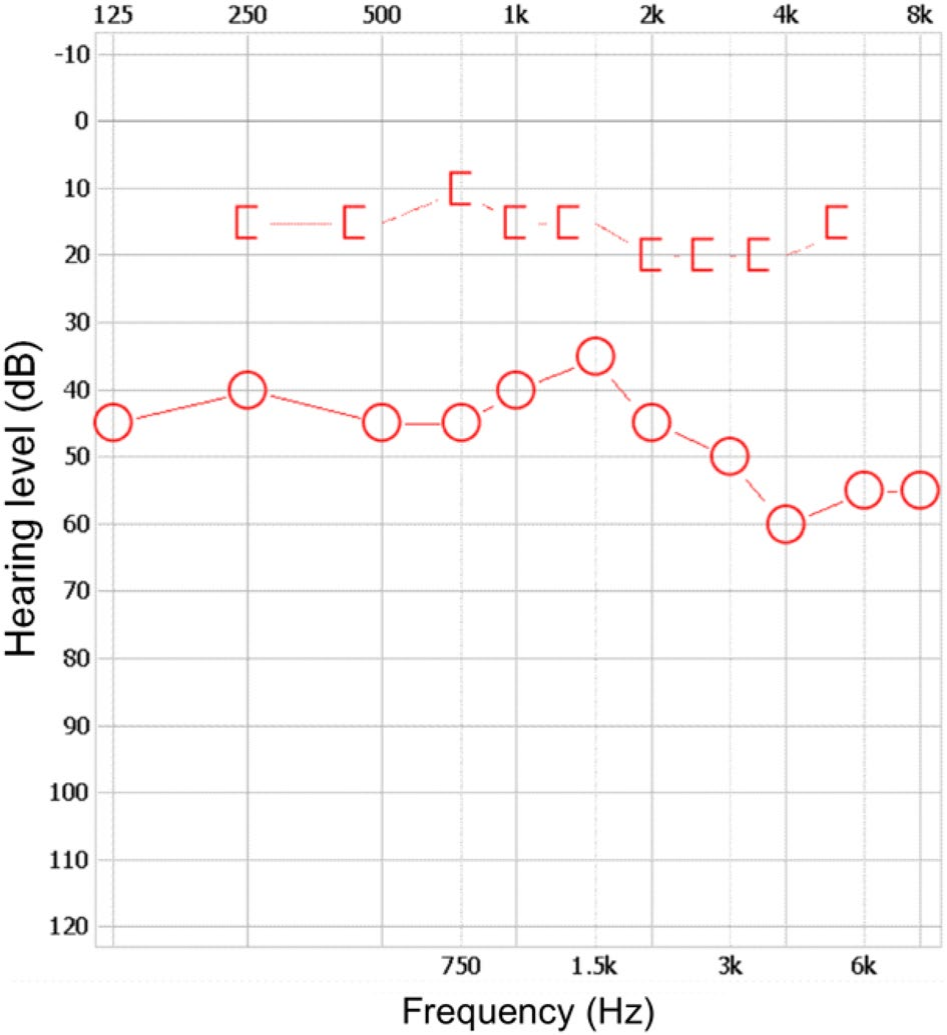
Two methods of representing tonal audiometry results: audiogram (left) and XML (right).

The results of pure-tone audiometry are commonly presented in the form of an audiogram, which is a graphical representation of how loud sounds must be at various frequencies for them to be audible. In addition to a graphical representation, audiology software generates XML files containing all information regarding tonal points that appear in the audiogram. The presented research uses XML files to analyse raw audiometry data. A sample audiogram, depicting masked right bone conduction ([) and non-masked right airconduction—(O) tresholds, with the corresponding XML file fragment containing the coordinates of consecutive tonal points is presented in Fig. 2.

The input data for a single measurement (one ear of one patient) consists of seven lists corresponding to air and bone conduction with hearing levels measured in decibels at frequencies of 125 Hz, 250 Hz, 500 Hz, 1000 Hz, 2000 Hz, 4000 Hz and 8000 Hz, respectively. It should be noted that two extreme frequencies (125 Hz and 8000 Hz) are not registered during bone conduction testing, which is why their values are set to null by default.

### Ethics declarations

This study was approved by the Committee on Research Ethics at Medical University of Gdańsk (IRB KB/23/2024). Prior to participating in the hearing test and this study, the subjects provided informed consent. All methods were carried out according to the relevant guidelines and regulations.

### Methodology

#### Data imbalance correction

An unbalanced training dataset, or in other words a dataset which does not evenly represent all possible classes, can significantly hinder the performance of machine learning models^28^. To prevent unintended outcomes from occurring when processing unbalanced data, a well-known system of class weight was employed^29^. This system permits the training procedure to account for the uneven distribution of classes by assigning different weights to the majority and minority classes. The objective is to penalise the model for the misclassification of the minority class by increasing its class weight and decreasing the class weight of the majority^30^. In the presented research, appropriate weight parameters were calculated and applied for each class during the training process.

#### Data normalisation

The process of data normalisation can aid in stabilising the gradient magnitude during training, particularly in the recurrent neural networks used in this study^31^. Experiments using several normalisation methods, such as linear normalisation, Robust Scaler and Max Abs Scaler, led to the selection of Z-score normalisation as the most effective^32^. Z-score normalisation refers to the process of normalising each value in a dataset so that the mean of all the values is 0 and the standard deviation is 1.

#### Network architecture

During a previous study^26^, several neural network architectures were evaluated in order to construct a binary classifier for normal and pathological hearing loss. The tested architectures included Multilayer Perceptron (MLP), Convolutional (CNN) and Recurrent (RNN) neural networks. A multi-stage investigation revealed that the RNN architecture performs best with this type of medical data. Over the course of that study, Recurrent neural networks (RNN), Gated Recurrent Units (GRU), and Long Short-Term Memory (LSTM) have been tested on a subset of the presented dataset. The final accuracy performance of RNN, GRU and LSTM was revealed to be 96.46%, 97.71%, and 98.12%, respectively. In addition, the LSTM model achieved an exemplary False Negative rate of 0.2%, which enabled its clinical application. Furthermore, these results have been corroborated by another study^33^, which investigated different neural network architectures for categorizing three forms of hearing loss, with an RNN-based model demonstrating the best performance out of the 11 models evaluated. These results are in line with the general notion that RNN architectures perform well with sequential or time-series medical data^34–36^, and LSTM in particular is well-known for successfully resolving problems with vanishing/exploding gradients^37^.

**Figure 3:**
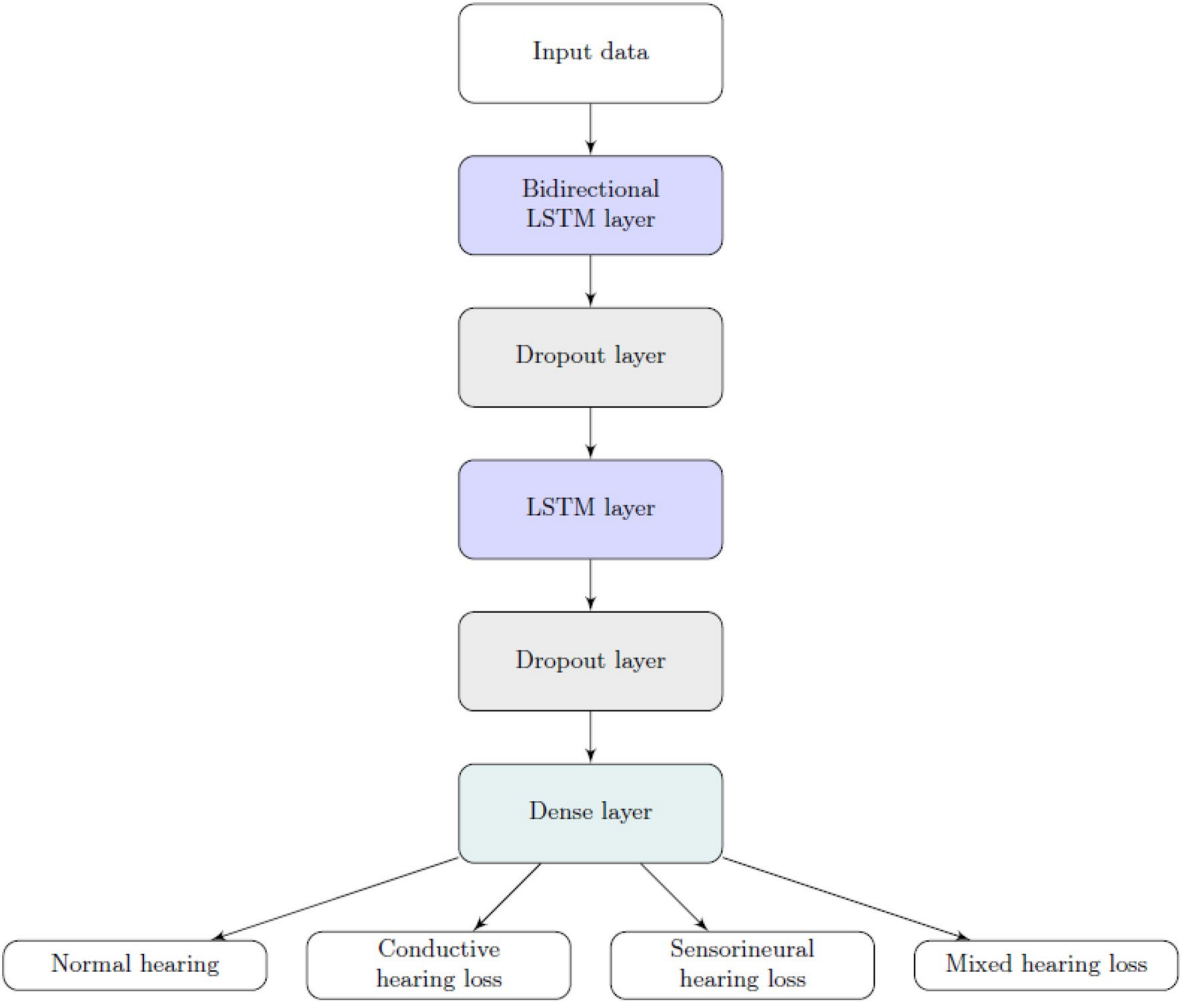
An overview of the proposed neural network architecture.

Due to the success of RNN-LSTM networks in the above-mentioned classifications, they have been selected for use in the presented study. The initial proposed multi-class solution involves the processing of input data from a single ear of a patient's audiometry test in the first LSTM layer. In the input data, time steps correspond to the tested frequencies, while air and bone conduction represent features in each time step. Afterwards, the number of nodes is reduced by a dropout layer, which helps prevent overfitting. The next steps consist of a similar sequence of LSTM and dropout layers. The model is completed by a dense layer with softmax activation function. After additional investigation and optimization, the initial model was modified by replacing the LSTM with a Bidirectional LSTM (Bi-LSTM)^38^ in the first layer. The Bi-LSTM is a variant of Bi-RNN that utilizes two basic LSTMs to analyse input time series in both forward and backward orientations. Utilizing data from both ways allows the model to detect patterns that could be overlooked when only using unidirectional LSTM. Thus, when considering pure tone audiometry data series, it can significantly improve classification accuracy. An overview of the proposed architecture is shown in Fig. 3.

#### Model evaluation

**Figure 4:**
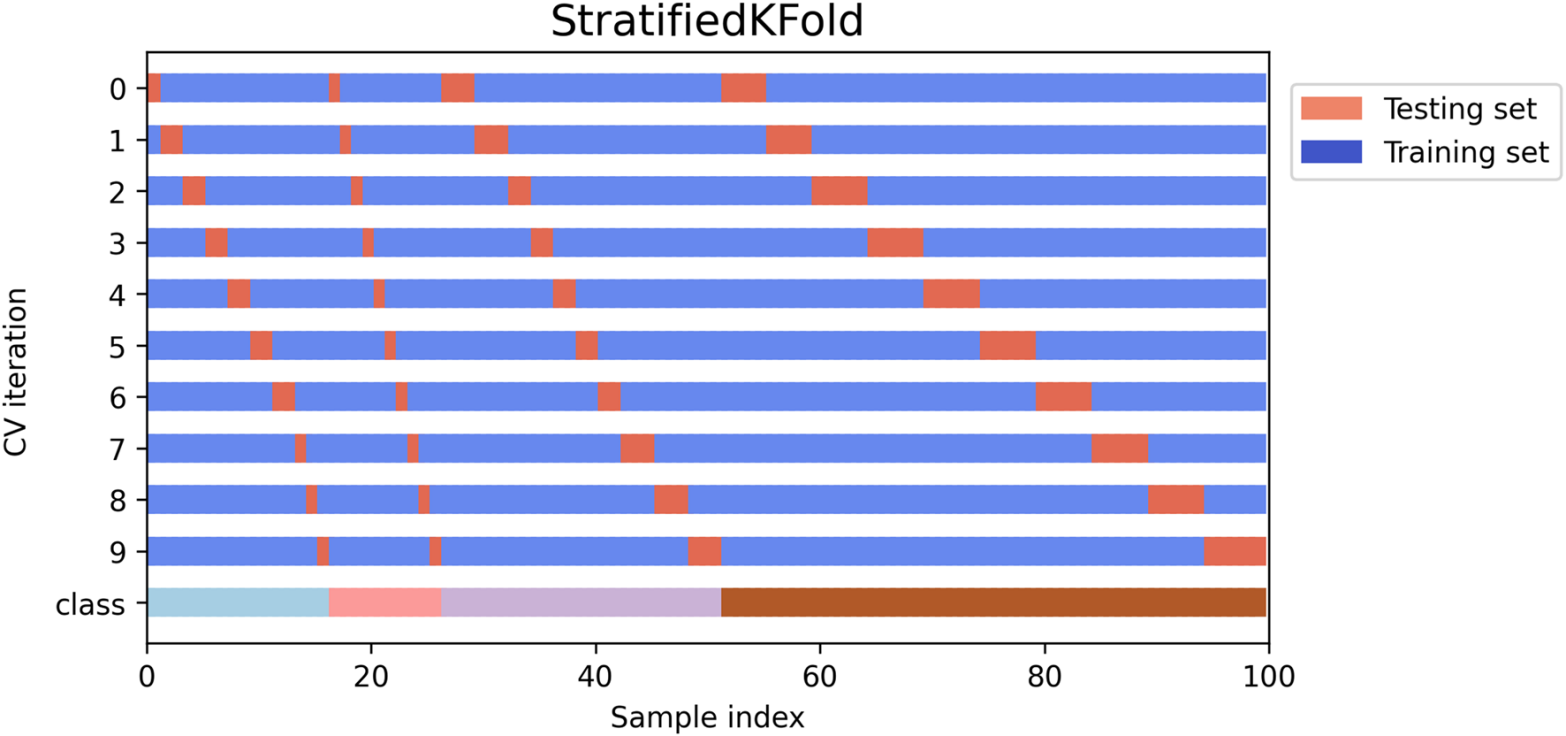
Schematic representation of a Stratified K-fold cross-validation, which uses proportional subsets of each class in every CV iteration.

Due to the aforementioned class imbalance, the model has been trained with the use of stratified K-fold cross validation (SKCV)^39^. K-fold Cross-Validation is the process of dividing a dataset into K folds and evaluating the model’s performance with new data. K represents the number of categories into which the data sample is divided. For instance, if the k-value is 10, it can be referred to as a tenfold cross-validation. At one point in the procedure, each fold serves as a test sample. SKCV is an extension of regular K-fold cross validation, which has been designed specifically for classification problems in which the ratio between the target classes is the same in each fold as it is in the entire dataset. In other words, the datasets are not distributed randomly into k-folds, but instead in a way that does not impact the sample distribution ratio across classes. Using stratified sampling rather than random sampling ensures that relative class frequencies are effectively maintained across each train and test fold. The behaviour of SKCV is represented graphically in Fig. 4.

**Figure 5:**
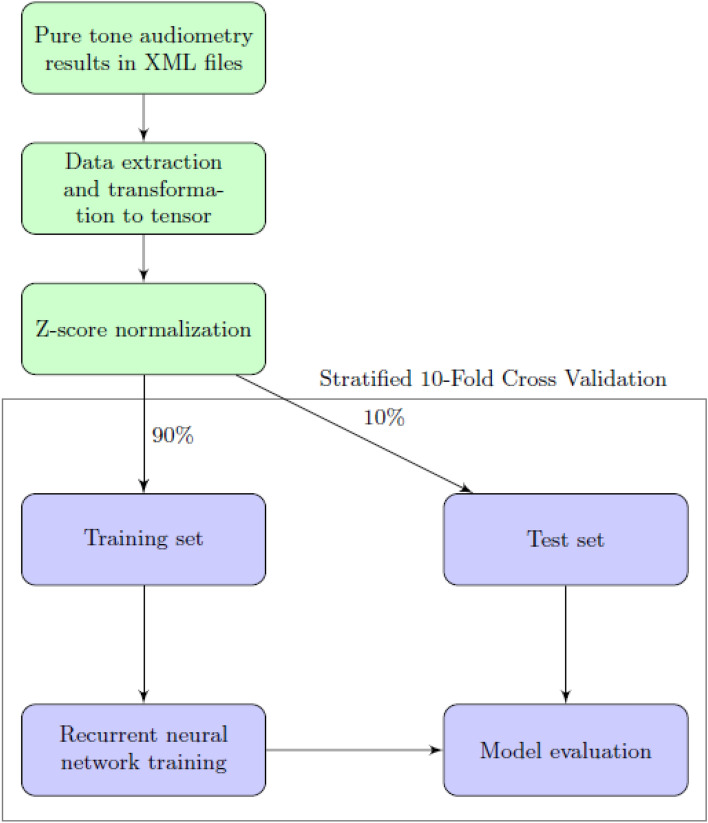
General workflow of the presented research.

Thus, the general workflow of the presented research is depicted in Fig. 5.

#### Metrics and statistical test

In the classification context, the performance of a classifier is typically evaluated by computing functions on the resulting confusion matrix. In essence, the confusion matrix represents the proportion of class samples that have been misclassified as other classes. For every class, there are four types of distinguishable parameters:

- True positive (TP), when positive predicted was true;
- True negative (TN), when negative predicted was true;
- False positive (FP), when positive predicted was false;
- False negative (FN), when negative predicted was false.

On the basis of these parameters, classification accuracy (1) is a common classification metric that computes the proportion of correctly classified test data relative to the total number of test data. In addition, precision (2) quantifies the proportion of positive class predictions that correspond to the positive class. In contrast, recall (3) computes the number of positive class predictions from all positive examples. Finally, F_1_ (4) provides a single score that addresses both precision and recall concerns in a single number.

1 $$Accuracy = \frac{TP + TN}{{TP + TN + FP + FN}}$$

2 $$Precision = \frac{TP}{{TP + FP}}$$

3 $$Recall = \frac{TP}{{TP + FN}}$$

4 $$F_{1} = 2 \cdot \frac{Precision \cdot Recall}{{Precision + Recall}}$$

The probability that a classifier provides more weight to the correct class than to the incorrect class is graphically presented in the form of Area Under the Curve (AUC). It is the area under a Receiver Operating characteristic Curve (ROC) that compares the true-positive rate to the false-positive rate by varying the decision threshold of the classifier.

In order to statistically compare performance of models the McNemar’s Test^40^ was used. The primary purpose of this test is to examine the disparities between two classifiers, specifically in relation to the instances where they made divergent predictions. The initial step entails performing calculations to determine the subsequent values:

- n00: number of items misclassified by both A and B;
- n01: number of items misclassified by A but not by B;
- n10: number of items misclassified by B but not by A;
- n11: number of items classified correctly by both A and B.

The null hypothesis states that the error rates of A and B, denoted as n01 and n10 respectively, are equal.

## Results

### Initial classification results

The applied stratified tenfold cross-validation of the proposed initial LSTM model yielded the following results: the average classification accuracy is 98.29% (+ /− 0.46%), the average precision is 98.30% (+ /− 0.47%), the average recall is 98.29% (+ /− 0.46%), and the average F_1_ score is 98.27% (+ /− 0.47%). Table 4 presents the detailed information of each phase of the SKCV procedure.

**Table 4:** Stratified tenfold cross validation score of proposed model.

Metrics	K1	K2	K3	K4	K5	K6	K7	K8	K9	K10
Accuracy (%)	97.67	98.34	97.87	98.87	97.94	98.14	98.34	99.14	97.87	98.74
Precision (%)	97.64	98.34	97.86	98.89	98.00	98.13	98.42	99.14	97.88	98.74
Recall (%)	97.67	98.34	97.87	98.87	97.94	98.14	98.34	99.14	97.87	98.74
F_1_ score (%)	97.63	98.33	97.85	98.86	97.87	98.10	98.35	99.13	97.87	98.74

**Figure 6:**
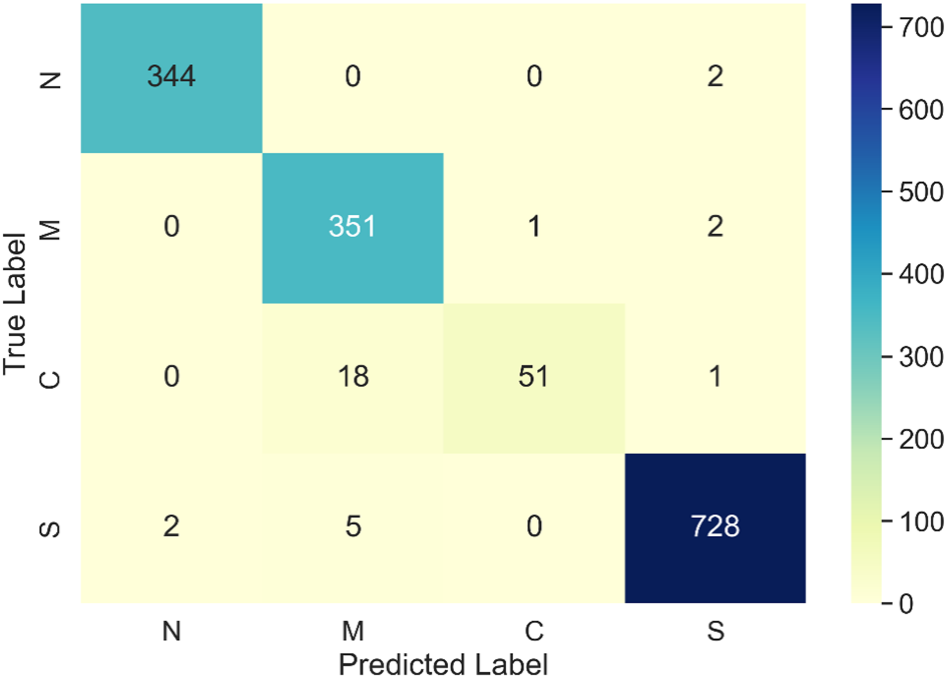
Confusion matrix of initial model.

The confusion matrix of the LSTM model is presented in Fig. 6. Normal hearing, mixed hearing loss, conductive hearing loss, and sensorineural hearing loss are represented by the N, M, C, and S indices, respectively.

### Final classification results

The applied stratified tenfold cross-validation of the proposed Bi-LSTM model yielded the following results: the average classification accuracy is 99.33% (+ /− 0.23%), the average precision is 99.32% (+ /− 0.33%), the average recall is 98.85% (+ /− 0.45%), and the average F_1_ score is 99.08% (+ /− 0.29%). Table 5 presents the detailed information of each phase of the SKCV procedure.

**Table 5:** Stratified tenfold cross validation score of proposed model.

Metrics	K1	K2	K3	K4	K5	K6	K7	K8	K9	K10
Accuracy (%)	99.27	99.53	99.27	99.14	99.00	99.34	99.40	99.60	99.73	99.00
Precision (%)	99.22	99.43	99.52	99.10	98.84	99.58	99.66	99.69	99.47	98.67
Recall (%)	98.35	99.50	98.09	98.42	99.30	98.92	98.79	98.90	99.47	98.76
F_1_ score (%)	98.76	99.46	98.78	98.75	99.07	99.25	99.22	99.29	99.47	98.71

Table 6 displays the precision, recall, and F_1_ score for each class of the proposed Bi-LSTM model.

**Table 6:** Comparison of each class’s precision, recall, and F_1_ score.

	Normal	Mixed hearing loss	Conductive Hearing Loss	Sensorineural hearing loss
Precision (%)	100.00	99.72	100.00	99.19
Recall (%)	99.42	99.15	98.57	99.86
F_1_ score (%)	99.71	99.43	99.28	99.53

**Figure 7:**
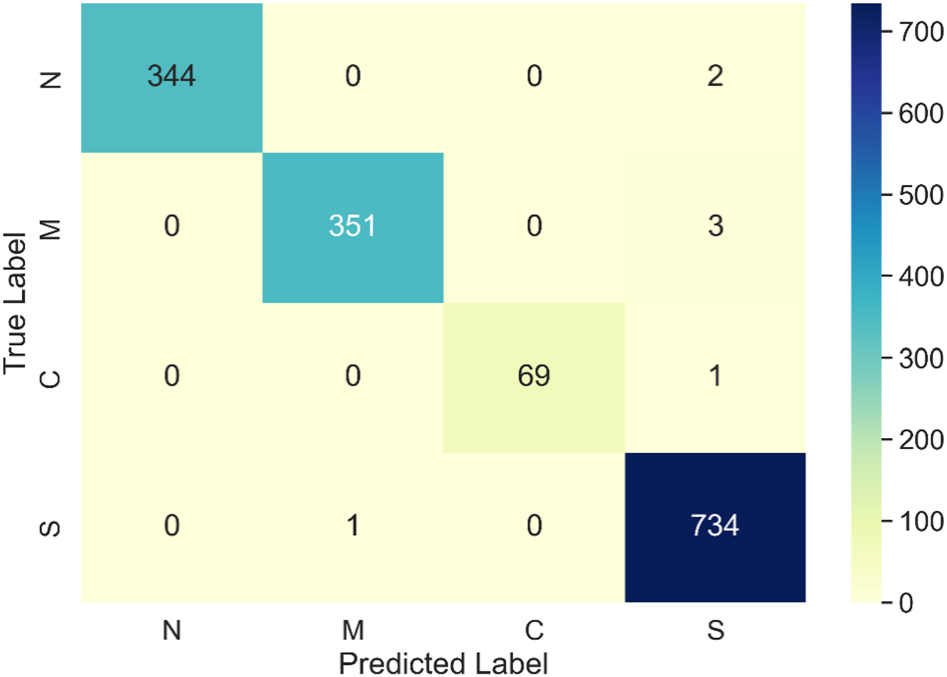
Confusion matrix of initial model.

The confusion matrix of the Bi-LSTM model is presented in Fig. 7.

### Comparison of different normalization methods

The issue of choosing an optimal neural network architecture as well as data normalization technique for the presented problem has been investigated in detail in^41^. The accuracy of both initial LSTM and proposed Bi-LSTM models using different normalization techniques is presented in Table 7.

**Table 7:** An analysis of the accuracy of LSTM and Bi-LSTM models using various normalizing approaches.

	LSTM	BI-LSTM
Linear normalization/Max–Min	97.21% (+/− 1.17%)	98.84% (+/− 0.37%)
Robust scaler	97.61% (+/− 0.75%)	99.04% (+/− 0.23%)
Max abs scaler	97.87% (+/− 1.54%)	99.27% (+/− 0.22%)
Z-score normalization	98.29% (+/− 0.46%)	99.33% (+/− 0.23%)

### Comparison with current state-of-the-art

The current state-of-the-art in raw audiometry data classification uses the C4.5 algorithm^42^ as a Decision Tree Classifier^9^. In order to facilitate a comparison, the C4.5 model was applied and evaluated on the presented dataset. Application of stratified tenfold cross-validation resulted in the following outcomes: the mean classification accuracy is 95.64% (+ /− 0.69%), precision 95.69% (+ /− 0.74%), recall 95.66% (+ /− 0.75%), F_1_ score 95.63% (+ /− 0.77%). Detailed results are provided below in Table 8.

**Table 8:** Stratified tenfold cross validation score of C4.5 model.

Hearing type	K1	K2	K3	K4	K5	K6	K7	K8	K9	K10
Accuracy (%)	96.21	95.67	94.15	96.54	96.15	95.95	95.28	95.41	96.14	94.88
Precision (%)	96.20	95.70	94.17	96.81	96.13	95.93	95.43	95.49	96.14	94.93
Recall (%)	96.21	95.68	94.15	96.76	96.15	95.95	95.28	95.41	96.14	94.88
F_1_ score (%)	96.17	95.67	94.07	96.76	96.12	95.92	95.27	95.39	96.12	94.83

**Figure 8:**
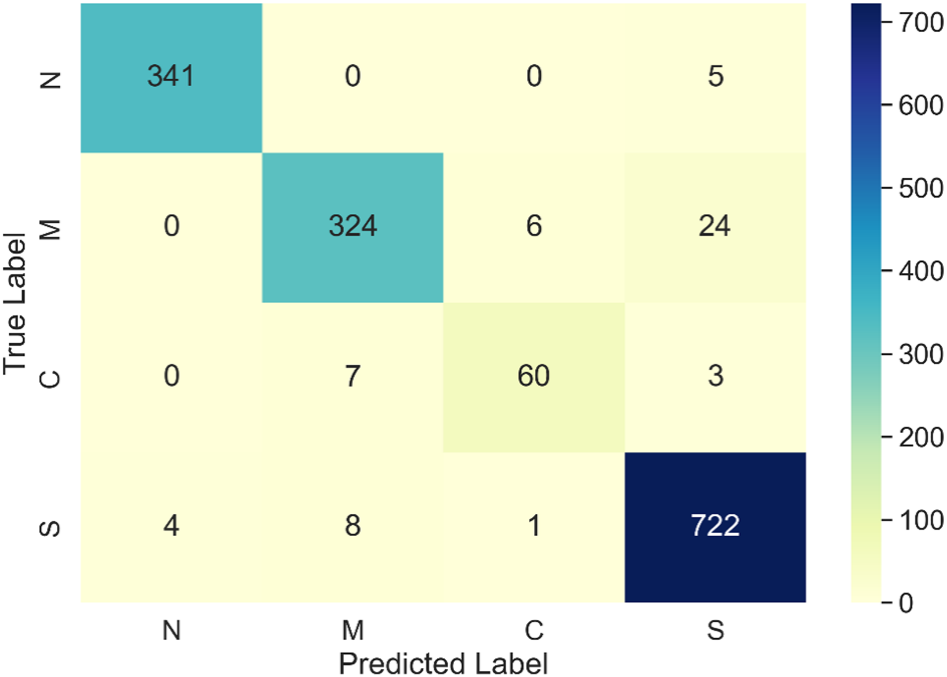
Confusion matrix of C4.5 model. The N, M, C, and S indices represent normal hearing, mixed hearing loss, conductive hearing loss, and sensorineural hearing loss, respectively.

The confusion matrix of the C4.5 model is presented in Fig. 8.

In order to statistically compare performance of the proposed BI-LSTM model with the C4.5 classifier we used McNemar’s Test. The significance level was determined at the value of $p=0.05$. The null hypothesis can be rejected in a two-tailed test if the calculated chi-square value $\left( {X^{2} } \right)$ exceeds the critical chi-square value $\left({X}^{2}\left(0.05\right)\right)$ at a significance level of $0.05$. The chi-square statistic yielded a value of 127.21, while the $p\text{-value}$ was determined to be 0.0000000001. The result provides evidence to reject the null hypothesis, indicating significant statistical difference between the two models.

**Figure 9:**
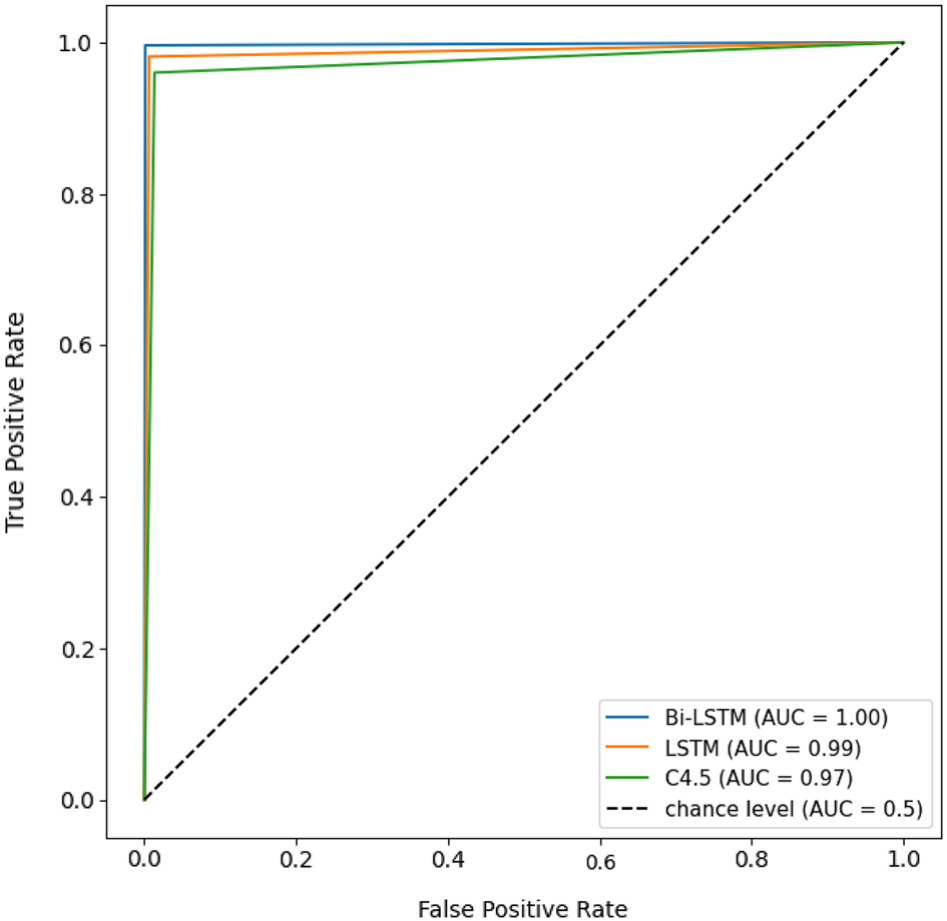
A ROC curve with the AUC parameter for each model.

The performance of solving a classification problem at different threshold settings is usually represented by the area under the receiver operating characteristic curve (AUC-ROC). The AUC-ROC is typically applicable to binary classification issues, however, the one-vs-all technique enables it to be extended to multiclass classification problems. The One-vs-the-Rest (OvR) multiclass strategy, also known as one-versus-all, involves computing a ROC curve for each class. In each stage, a specific class is viewed as the positive class, while the remaining classes are viewed as the negative class in the majority. The micro-averaged ROC curves with AUC parameters of models is shown in Fig. 9.

## Discussion

The initial LSTM model satisfactorily classified normal hearing (N), sensorineural (S), conductive (C), and mixed hearing loss (M) in terms of average accuracy of 98.29% (+ /− 0.46%) based on stratified tenfold cross validation. The average accuracy is superior than the existing state-of-the-art. However, the results displayed in Fig. 6 render the model unsuitable for clinical applications. The main concern is the presence of false negatives in individuals with normal hearing, which may lead to the patient being at risk of not obtaining appropriate medical treatment. In other words, the classification precision in the case of normal hearing ought to be 100%. That said, this requirement is not met as there are four instances where sensorineural hearing loss was incorrectly classified as normal hearing. Therefore, more efforts have been made to enhance the model. Several different architectures were investigated, but only one variant of the traditional LSTM, the Bidirectional LSTM, yielded improved results. This observation is supported by a number of published studies in which authors demonstrate that bidirectional LSTM models outperform conventional LSTM models. This insight is apparent in research on natural language processing^43,44^, but it applies to other fields as well^45–48^. An example of an application outside of natural language processing is an article that concentrates on forecasting the spread of the COVID-19 pandemic using a Bi-LSTM model on time series data^48^. Out of the fifteen models tested, Bi-LSTM achieved the highest performance, exceeding that of LSTM and other RNN variants. This potential to further improve LSTM classification results motivated us to evaluate the performance of Bi-LSTM on our dataset.

Based on stratified tenfold cross validation, the proposed Bi-LSTM model successfully classified normal hearing (N), sensorineural hearing (S), conductive hearing (C), and mixed hearing loss (M) with an average accuracy of 99.33%. Beginning at 99.00 and ending at 99.73, the accuracy remained stable, with standard deviation equal to 0.23%. Precision, recall, and F_1_ score share similar characteristics with regard to accuracy. Table 6 revealed a diversity of outcomes, with performance parameters broken down by individual classes. The classification performance of normal hearing, mixed hearing loss and sensorineural hearing loss has shown to be notably high. In contrast, cases of conductive hearing loss have been classified with lower accuracy, particularly in terms of recall. The precise definition of recall for conductive hearing loss is the ratio of correctly predicted cases of conductive hearing loss (69) to actual number of conductive hearing loss cases (69 + 1 = 70), which is approximately 98.57%. There are a few explanations for this behaviour. To begin with, only 4.37% of the dataset represents conductive hearing loss (Table 2). This implies that any misclassification will have a greater effect on statistical calculations that take true positive examples into consideration. Furthermore, regardless of the weighting method used in the training process, NN has a lower chance of learning patterns from smaller amounts of data, as demonstrated by^49^. The lower number of conductive hearing loss samples in the used dataset is caused by the rules of patient treatment employed by medical institutes. In particular, conductive hearing loss is usually caused by pathology along the root of the ear, which essentially means that an object is blocking the air canal. This type of problem is typically diagnosed with an otoscope during the initial patient examination, therefore nullifying the need for conducting a pure-tone audiometry test. Currently there is no practical way to alleviate this problem, as performing audiometry tests on patients who can be diagnosed by simpler methods is not financially viable.

The results presented in Table 7 demonstrate that the selection of the optimal normalizing procedure considerably affects the final accuracy value. Irrespective of the model used, Z-score normalization proved to be the most effective strategy for scaling features, closely followed by Max Abs Scaler. The application of linear normalization (Max–Min) resulted in a decrease in the degree of accuracy, particularly the loss of over one percentage point in the LSTM model and around half a percentage point in the Bi-LSTM model.

The proposed solution significantly outperformed the current state-of-the-art in raw audiometry data classification, held by the C4.5 Decision Tree (DT-J48) method proposed by Elbaşı and Obali^9^. Application of the C4.5 classifier to the presented dataset demonstrated a level of accuracy which is in line with the one reported by the original publication, with a mean value of 95.64% and a standard deviation of 0.69%. This effectively makes the state-of-the-art model around 4 percentage points inferior compared to the proposed Bi-LSTM model. The dissimilarity is also evident in the confusion matrices of both models. Specifically, there is a notable distinction in the misclassification of mixed hearing loss (M) as sensorineural hearing loss (S). The C4.5 model exhibits 24 instances of this misclassification, whereas the Bi-LSTM model demonstrates only 3 instances. Furthermore, there are four cases in which hearing loss was misclassified as normal hearing in the C4.5 model. It is worth noting that such misclassifications were not observed in the Bi-LSTM model. The distinction between C4.5 and BI-LSTM is also evident in the ROC-AUC curves shown in Fig. 9. The C4.5 model with an AUC of 0.97 performed less effectively compared to the Bi-LSTM model with an AUC of 1.0. Finally, the findings obtained by McNemar's Test indicate a statistically significant difference in the classification outcomes between the C4.5 model and the Bi-LSTM model.

In general terms of the audiogram classification problem, the overall accuracy of the presented model (99.33%) exceeds the most performant of the existing approaches to hearing loss classification, presented by Crowson et al. for raster data^22^, which is 97.5%. When compared directly, the difference in accuracy may not seem very large, however it should be noted that it was obtained on a significantly larger dataset (1007 samples in Crowson et al.^22^ versus 15,046 in this study), which ensured proper variation of training as well as validation data and guaranteed that the obtained classification results are not overly optimistic. In machine learning, the larger and more diverse the dataset, the better it is for discovering general patterns, particularly in medical applications where specific cases occur infrequently but must be evaluated correctly by NN. Classification of hearing loss in audiograms is typically based on frequencies between 0.5 and 4 kHz, but hearing loss can also be detected in the upper pitch range of 4 to 8 kHz^13^. Consequently, it is crucial to train on a sufficient number of examples to illustrate these specific audiometry challenges. It should also be noted that the work presented by Crowson et al. has been based on interpretation of raster audiogram images. As these images are the outcome of pure-tone audiometry tests, working with them is the intuitive approach. Unfortunately, the majority of audiogram images are generated by specialised software provided by different hardware vendors. While the symbols appearing in audiograms are standardised by the American Speech-Language-Hearing Association^50^, there are no strict rules regarding other aspects of creating audiograms. As a result, images from different sources can have a large variety of differences, ranging from small details such as variance in colour of plots and size of measurement point indicators, to changes which can significantly impede the performance of an automated classifier, such as placing the test results from both ears on a single plot. As a result, image-trained models such as those presented by Crowson et al.^22^ and Charih et al.^20^ will function properly only with specific sources of audiometry data. In comparison, the classifier developed during the presented study works on raw audiometry data, allowing it to bypass vendor-specific issues with data representation.

Moreover, every system that will be used in clinical settings must meet extremely stringent requirements, in order to ensure that it does not pose undue risk to patients. In this context, it is not only important that the developed classifier achieves a high level of overall accuracy, but the types of errors it may be prone to make are also crucial. In the context of the presented work, the most dangerous scenario is when a patient with hearing loss is misclassified as having normal hearing, which can result in them not receiving proper medical care. Therefore, a secondary goal of the presented research has been to eliminate this type of error. The results of this endeavour are visible in the confusion matrix of the presented model (Fig. 7). As it can be seen in Fig. 7, there are no instances in which conductive hearing loss, mixed hearing loss, or sensorineural hearing loss are categorised as normal hearing by the developed model. While the developed model is not completely error-free, its potential practical application should not put patients at risk. This is a significant step-up from the current state-of-the-art C4.5 model proposed by Elbaşı and Obali^9^, which yielded five instances of misclassification that may result in patients not obtaining appropriate medical care. Moreover, while both classifiers exhibit instances when individuals with normal hearing were erroneously identified as having sensorineural hearing loss, the proposed model exhibits a lesser number of this sort of error compared to the state-of-the-art, with 2 errors in the Bi-LSTM model as opposed to 5 errors in the C4.5 model. However, this type of error is less significant, as it would result in the patient being directed to a qualified audiologist who would rectify the mistake.

## Conclusion

This paper presents a Bi-LSTM-based model for classification of raw audiometry data into normal hearing and three types of hearing loss. The developed solution advances the classification of hearing loss types beyond the current state-of-the-art in several areas. First, the achieved classification accuracy (99.33%) is superior to that presented in current state-of-the-art in raw audiometry data classification, presented by Elbaşı and Obali^9^, which achieved 95.5%. The findings obtained from the comparative analysis between the C4.5 model proposed by Elbaşı and Obali and the Bi-LSTM model presented in this study using the same dataset indicate that the Bi-LSTM model exhibits significantly higher accuracy and does not produce any errors that could negatively impact patient health.

Secondly, the proposed solution also managed to outperform the current state-of-the-art in raster audiogram classification presented by Crowson et al.^22^ which achieves 97.5% accuracy.

Thirdly, the presented research was conducted on 15,046 audiometry test result samples, which is nearly 15 times larger than the largest dataset produced to date in terms of hearing loss type, which consists of 1007 audiograms and was established by Crowson et al.^22^. The high variety and representativeness of the used dataset ensures that the reliability of the obtained results also constitutes an improvement to the state-of-the-art.

Finally, working with raw audiometry data allows for a more flexible implementation in clinical settings. In contrast to the approach presented e.g. by Crowson et al.^22^, the presented method is not limited to working with audiogram images produced by specific sources.

This being said, there are a few limitations associated with this study. Using an unbalanced dataset with only 4.37% instances of conductive hearing loss results in a lower value of F_1_ score compared to other classes. Moreover, working with raw audiometry data means that the classifier can only be used in medical facilities, as patients are generally only presented with audiogram images of their test results. In consequence, further work is needed to integrate the presented model with an accurate audiogram image parser in order to make it more broadly available to patients as well as physicians.

Overall, the presented results suggest that the developed NN-based audiometry data classifier can be applicable to clinical practice, either in the form of a classification system for general practitioners or a support system for professional audiologists. Moreover, the model can work with all hardware systems that generate text results of audiometry tests. By allowing general practitioners to classify the results of pure tone audiometry tests, the developed model may help to significantly reduce the caseload of audiology specialists. In addition, the proposed solution gives professional audiologists access to an AI decision support system that has potential to reduce their workload while also increasing diagnostic precision and decreasing human error.

## Data Availability

The datasets analysed during the current study are not publicly available due to the confidentiality restrictions imposed by the approved ethics of study but are available from the corresponding author on reasonable request.
